# Dynamic ^11^C-Methionine PET-CT: Prognostic Factors for Disease Progression and Survival in Patients with Suspected Glioma Recurrence

**DOI:** 10.3390/cancers13194777

**Published:** 2021-09-24

**Authors:** Maria Vittoria Mattoli, Gianluca Trevisi, Valentina Scolozzi, Amedeo Capotosti, Fabrizio Cocciolillo, Irene Marini, Valerio Mare, Luca Indovina, Massimo Caulo, Antonella Saponiero, Mario Balducci, Silvia Taralli, Maria Lucia Calcagni

**Affiliations:** 1 Department of Neurosciences, Imaging and Clinical Sciences, University “G. d’Annunzio” of Chieti, 66100 Chieti, Italy; mvittoriamattoli@yahoo.it (M.V.M.); caulo@unich.it (M.C.); 2 Neurosurgical Unit, Presidio Ospedaliero Santo Spirito, 65124 Pescara, Italy; trevisi.gianluca@gmail.com; 3 UOC di Medicina Nucleare, Dipartimento di Diagnostica per Immagini, Radioterapia Oncologica ed Ematologia, Fondazione Policlinico Universitario A. Gemelli IRCCS, 00168 Roma, Italy; fabrizio.cocciolillo@guest.policlinicogemelli.it (F.C.); silvia.taralli@policlinicogemelli.it (S.T.); marialucia.calcagni@unicatt.it (M.L.C.); 4 Fondazione Policlinico Universitario A. Gemelli IRCCS, 00168 Roma, Italy; amedeo.capotosti@policlinicogemelli.it (A.C.); luca.indovina@policlinicogemelli.it (L.I.); 5 Nuclear Medicine and Radiometabolic Unit, Istituto Scientifico Romagnolo per lo Studio e la Cura dei Tumori (IRST) IRCCS, 47014 Meldola, Italy; irene.marini88@gmail.com; 6 Università Cattolica del Sacro Cuore, 00168 Roma, Italy; valerio.mare01@icatt.it; 7 Department of Radiology, University “G. d’Annunzio” of Chieti, 66100 Chieti, Italy; 8 UOSD di Oncologia, Dipartimento di Oncologia, Presidio Cassia-S. Andrea-ASL RM1, 00100 Rome, Italy; antonella.saponiero@aslroma1.it; 9 UOC Radioterapia Oncologica, Dipartimento di Diagnostica per Immagini, Radioterapia Oncologica ed Ematologia, Fondazione Policlinico Universitario A. Gemelli IRCCS, 00168 Roma, Italy; mario.balducci@policlinicogemelli.it; 10 Dipartimento Universitario di Scienze Radiologiche ed Ematologiche, Università Cattolica del Sacro Cuore, 00168 Roma, Italy

**Keywords:** ^11^C-methionine, PET-CT, dynamic acquisition, glioma, prognosis

## Abstract

**Simple Summary:**

Recurrence after initial treatments is an expected event in glioma patients, particularly for high-grade glioma, with a median progression-free survival of 8–11 weeks. The prognostic evaluation of disease is a crucial step in the planning of therapeutic strategies, in both the primary and recurrence stages of disease. The aim of our retrospective study was to assess the prognostic value of ^11^C-methionine PET-CT dynamic and semiquantitative parameters in patients with suspected glioma recurrence at MR, in terms of progression-free survival and overall survival. In a population of sixty-seven consecutive patients, both static and kinetic analyses provided parameters (i.e., tumour-to-background ratio and SUVmax associated with time-to-peak, respectively) able to predict both progression-free and overall survival in the whole population and in the high-grade glioma subgroup of patients. Dynamic ^11^C-methionine PET-CT can be a useful diagnostic tool, in patients with suspicion of glioma recurrence, able to produce significant prognostic indices.

**Abstract:**

Purpose: The prognostic evaluation of glioma recurrence patients is important in the therapeutic management. We investigated the prognostic value of ^11^C-methionine PET-CT (MET-PET) dynamic and semiquantitative parameters in patients with suspected glioma recurrence. Methods: Sixty-seven consecutive patients who underwent MET-PET for suspected glioma recurrence at MR were retrospectively included. Twenty-one patients underwent static MET-PET; 46/67 underwent dynamic MET-PET. In all patients, SUVmax, SUVmean and tumour-to-background ratio (T/B) were calculated. From dynamic acquisition, the shape and slope of time-activity curves, time-to-peak and its SUVmax (SUVmax_TTP_) were extrapolated. The prognostic value of PET parameters on progression-free (PFS) and overall survival (OS) was evaluated using Kaplan–Meier survival estimates and Cox regression. Results: The overall median follow-up was 19 months from MET-PET. Recurrence patients (38/67) had higher SUVmax (*p* = 0.001), SUVmean (*p* = 0.002) and T/B (*p* < 0.001); deceased patients (16/67) showed higher SUVmax (*p* = 0.03), SUVmean (*p* = 0.03) and T/B (*p* = 0.006). All static parameters were associated with PFS (all *p* < 0.001); T/B was associated with OS (*p* = 0.031). Regarding kinetic analyses, recurrence (27/46) and deceased (14/46) patients had higher SUVmax_TTP_ (*p* = 0.02, *p* = 0.01, respectively). SUVmax_TTP_ was the only dynamic parameter associated with PFS (*p* = 0.02) and OS (*p* = 0.006). At univariate analysis, SUVmax, SUVmean, T/B and SUVmax_TTP_ were predictive for PFS (all *p* < 0.05); SUVmax_TTP_ was predictive for OS (*p* = 0.02). At multivariate analysis, SUVmax_TTP_ remained significant for PFS (*p* = 0.03). Conclusion: Semiquantitative parameters and SUVmax_TTP_ were associated with clinical outcomes in patients with suspected glioma recurrence. Dynamic PET-CT acquisition, with static and kinetic parameters, can be a valuable non-invasive prognostic marker, identifying patients with worse prognosis who require personalised therapy.

## 1. Introduction

Gliomas represent about 50% of primary brain tumours, with the majority of them presenting as or evolving to a high-grade glioma (HGG). Recurrence after initial treatments is an expected event in glioma patients, particularly for HGG with a median progression-free survival of 8–11 weeks [1]. Despite the advances of several diagnostic tools and a multidisciplinary treatment approach with standard therapies, the prognosis of glioma patients, particularly of HGG, is generally poor. The prognostic evaluation of disease is a crucial step in the planning of therapeutic strategies, in both the primary and recurrence stages of the disease.

It is well known that the main clinical and molecular prognostic factors for glioma are age, histopathologic type, presence of neurological deficits, Karnofsky performance status score, isocitrate dehydrogenase (IDH) status and 1p/19q co-deletion, along with tumour size, World Health Organisation (WHO) grading and surgical aggressiveness [2,3]. Structural MR gives additional prognostic information, including preoperative tumour characteristics, such as sub-ependymal spreading and post-operative extent of resection [4,5,6].

Positron emission tomography (PET) is a functional molecular technique able to early detect pathophysiological changes in gliomas that usually occur before the morpho-structural changes detected by structural imaging [7,8]. PET-CT with amino-acid radiotracers, including carbon-11 methionine (^11^C-MET), fluorine-18 fluoroethyltyrosine (^18^F-FET) and fluorine-18 fluorodihydroxyphenylalanine (^18^F-DOPA), is a well-established tool in the evaluation of glioma patients in different phases of disease, including prognostic evaluation [9,10,11,12,13,14,15]. The prognostic value of semiquantitative PET parameters, such as tumour maximum uptake/reference background mean uptake (T/B) and metabolic tumour volume, has been proven for different PET amino-acid tracers [16,17]. Despite logistic disadvantages owing to the shorter half-life of ^11^C compared to ^18^F, the usefulness of static ^11^C-MET parameters in the evaluation of prognosis has been increasingly explored in both primary and recurrence glioma patients [18,19,20,21,22,23].

In addition to static indices, dynamic amino-acid PET parameters, such as time-to-peak (TTP) and the slope of the time-activity curve (TAC), have been explored in different stages of the disease and in the evaluation of prognosis. In particular, dynamic ^18^F-FET indices have been proven to be prognostic factors in newly diagnosed gliomas [24,25,26] and in recurrence [27,28]. Recently, Zagori et al. [29] explored the usefulness of dynamic ^18^F-DOPA parameters in the prognostication of patients with suspected glioma recurrence, failing to prove the definitive primacy of these indices compared to static ones. Dynamic ^11^C-MET parameters have been studied in the differential diagnosis of brain tumours [30,31] to characterise glioblastoma with an oligodendroglial component [32] and to distinguish high-grade from low-grade glioma (LGG) [33], reaching conflicting results. Since differences in uptake mechanism are present among amino-acids radiotracers, the findings observed for dynamic ^18^F-FET and ^18^F-DOPA in the prognostic evaluation cannot directly be translated to ^11^C-MET.

To the best of our knowledge, no data are evaluable on the prognostic role of dynamic ^11^C-MET PET parameters in glioma patients, including recurrent ones. The aim of our study was to investigate the prognostic value of kinetic parameters extracted from dynamic ^11^C-MET PET-CT in patients with suspected recurrence of glioma, in terms of progression-free survival and overall survival.

## 2. Materials and Methods

### 2.1. Patients

We retrospectively analysed the ^11^C-methionine PET-CT scans (MET-PET) of 67 consecutive patients (40 males/27 females; mean age: 48 ± 15 years) with a history of previously treated glioma who were referred between December 2013 and December 2018 to our institution for suspected recurrence at MR. A retrospective review of medical records of all patients was performed in order to collect demographic, clinical and radiological data, including details on previous surgical procedures and adjuvant treatments.

All patients had a surgical procedure at first presentation of the tumour, with histologically confirmed diagnosis of low-grade glioma (WHO grade I or II) in 30/67 (44.8%) patients and of HGG (WHO grade III or IV) in 37/67 (55.2%). First-line adjuvant treatment with chemotherapy and/or radiotherapy was administered in 54/67 (80.6%) patients according to the latest guidelines [34]. The patients’ demographic characteristics, tumour histopathological features and first-line and second-line treatments are presented in Table 1.

In all patients, MET-PET was performed during follow-up since the structural MR performed at the referring centre was suspected but could not definitively prove whether a tumour progression or recurrence, based on Response Assessment in Neuro-Oncology (RANO) criteria, had occurred. In all cases, at least the following MR sequences were performed: T1 and T2 weighted images, fluid attenuated inversion recovery images (FLAIR), diffusion weighted images (DWI) and T1 sequences after gadolinium infusion. However, regardless of the MET-PET result, the definitive assessment of disease progression was based on the following evidence: biopsy/surgery, subsequent MR and/or ^11^C-methionine PET-CT studies or a significant worsening of patient’s clinical status related to brain pathology.

Institutional review board approval has been preliminary obtained for the use of data stemming from standard clinical practice for research purposes, as no additional intervention was planned (retrospective observational study). All study-related procedures have been performed according to the Declaration of Helsinki.

### 2.2. PET-CT Imaging: Acquisition Protocol

All patients fasted for at least 6 h and underwent brain ^11^C-methionine PET-CT acquisition 15 ± 5 days after structural MR. All examinations were performed using Biograph mCT (Siemens Medical Solutions USA, Inc., Knoxville, TN) PET-CT scanner systems. Each patient was placed in a supine position in the scanner with the brain in the field of view with a head holder to maintain the head in the same position.

Twenty-one out of 67 (44.7%) patients underwent a 10 min static brain PET-CT acquisition, obtained 10 min after injection of ^11^C-methionine (ranging from 325 to 401 MBq; 6.8 MBq/kg body weight). In the remaining 46/67 (68.6%) patients, a 3D brain dynamic list-mode acquisition was started at ^11^C-methionine injection (ranging from 370 to 555 MBq, 7.0 MBq/kg body weight), which lasted 20 min. Images were reconstructed with the following framing: 9 frames of 5 s each, 1 frame of 30 s, 1 frame of 45 s, 1 frame of 3 min, and 3 frames of 5 min each. A static image from data collected between 10 and 20 min after injection was also reconstructed. Before PET acquisition, a low-dose CT scan was performed (tube current 75 mA, voltage 120 kV, pitch 0.55, rotation time 1 s) for both brain attenuation correction of the PET emission data and for morphological information. PET data were corrected for random events, dead time and attenuation.

### 2.3. PET-CT Imaging: Data Analysis

PET and MR images were co-registered using dedicated software (MIM Maestro V.6.9.6) and the images were analysed by two nuclear medicine physicians (M.V.M. and V.S.) with PET-CT experience in the brain. The fusion results were inspected and adapted based on anatomical landmarks, if necessary.

The semi-quantitative evaluation of static MET-PET images was performed in all 67 patients using regions of interest (ROIs). In case of visually positive MET-PET, a ROI was manually drawn over the area of highest tracer accumulation (tumour ROI). In case of visually negative MET-PET, a ROI was manually drawn on MR images (T1 contrast enhancement and/or FLAIR) and subsequently transferred on PET images. The uptake of the unaffected brain tissue was determined by a circular ROI of 20 mm^3^ placed in the contralateral hemisphere in an area of normal-appearing brain tissue as background reference (background ROI) [35]. Tracer accumulation in the ROIs was expressed as standardised uptake value (SUV) by dividing the radioactivity (kBq/mL) in the ROI by the radioactivity injected per gram of body weight. Maximum SUV (SUVmax) and mean SUV (SUVmean) were noted for both ROIs. Furthermore, the tumour-to-background ratio (T/B) of MET-PET was calculating by dividing the SUVmax of the tumour ROI by the SUVmean of the background ROI.

In patients with dynamic PET acquisition (*n* = 46), each tumour ROI was copied from the static acquisition to the entire set of dynamic PET images, and individual time–activity curves (TAC) of the SUVmean of the lesion were generated. According to the course of the TAC, two different types of TAC were observed in the study population: (1) lesion with increasing uptake over time (type A), with SUV with a rapid increase in the early phase and a slow increase in the late phase; (2) tumour with decreasing uptake over time (type B), with SUV showing a rapid increase in the early phase and a slow decrease in the late phase [30]. The trend of the TACs was also assessed from 75 s post injection by fitting with a logarithmic curve for type A patients and with a divided rational curve for type B patients. The first derivative of the fitting curve gives the SUV variation rate per second (slope, [1/s]).

Finally, two parameters were extracted from dynamic data: (1) the time-to-peak (TTP) of the entire dynamic acquisition, defined as the time (in seconds) from the start of the dynamic acquisition up to the maximum value of SUV and (2) its relative SUV (SUVmax_TTP_) [33]. In the case of steadily increasing MET uptake during the study, the end of study was defined as TTP.

### 2.4. Statistical Analysis

Patients were followed-up until death or until December 2018 in still-living patients. To the aim of this study, progression-free survival (PFS) and overall survival (OS) were calculated using the date of the MET-PET as starting point. Indeed, PFS was defined as the time (months) from the date of the MET-PET to the date of tumour recurrence/progression documented by biopsy/surgery or the evidence of tumour growth on subsequent conventional MR according to RANO criteria [9,36], or on additional ^11^C-methionine PET-CT scan or a not otherwise explained and not responsive to steroids reduction of at least 20% in the Karnofsky performance score (KPS) during follow-up. The OS was defined as the time (months) from the MET-PET to death. For patients who had not experienced recurrence or death at the time of last follow-up, PFS and OS were censored at the date of last follow-up. Patients not defined by the cause of death query were censored for further calculations by the date of the last documented visit at the hospital/contact with referring physician.

Continuous data are expressed as means ± standard deviation (SD) or median and interquartile range. Independent samples Student’s *T* test or Mann–Whitney rank-sum test were used to compare quantitative variables. The diagnostic performance of MET-PET, as determined by SUVmax, SUVmean, T/B, TTP, SUVmax_TTP_ and slope of TAC, was assessed by additional receiver operating characteristic (ROC) curve analysis using PFS at 6 months and OS at 12 months as reference. The decision cut-off was considered optimal when the product of paired values for sensitivity and specificity reached its maximum. As measure of the diagnostic quality of the test, the area under the ROC curve (AUC), its standard error and the level of significance were determined.

PFS and OS were calculated from the MET-PET date using the Kaplan–Meier method and compared using a logrank test, as univariate survival analysis. A Cox proportional hazards regression model was used to identify the independent predictors of PFS and OS, and estimated hazard ratios and 95.0% confidence intervals (CIs) were calculated. Variables with a *p* value less than 0.05 in the univariate analysis were included in the multivariate analysis.

A subgroup analysis was conducted for histologically proven HGGs, including the HGGs at primary diagnosis (*n* = 37) and those initial LGGs with a histologically proven progression towards a higher tumour grade at second surgery (*n* = 3). The statistical analysis was performed using MedCalc software (version 11.6; Broekstraat, Mariakerke, Belgium).

## 3. Results

### 3.1. Patient Population

The clinical characteristics of the examined population (*n* = 67) are reported in Table 1. The interval between primary tumour diagnosis and MET-PET was 4.9 years ± 6.0 years. After a median follow-up from MET-PET of 19 months (range: 7–37 moths), 46/67 patients (68.7%) had a recurrence, of whom 38/46 were within 6 months and 8/46 were after 6 months from PET. Recurrence was diagnosed with MR in 29/46 patients and with histopathology in 13/46 (2 LGG, 11 HGG); in 4/46 patients, disease progression was suggested by a rapid, dramatic worsening of clinical condition with a reduction in the KPS ≥ 20% and death within 4 weeks in 3 cases and 3 months in another patient. Median PFS from MET-PET evaluation to confirm the recurrence was 1 month (range: 1–21 months). According to primary WHO grade, recurrence occurred in 1/1 (100%) grade I glioma, in 17/29 (58.6%) grade II, in 8/13 (61.5%) grade III, and in 20/24 (83.4%) grade IV glioma. Thirty-five out of 67 patients underwent second-line treatments, of whom 13 consisted of surgery.

At the time of the last follow-up, 23/67 (34.3%) patients had died, of whom 16/23 had died within 12 months from PET: 2/29 (6.9%) grade II, 4/13 (30.7%) grade III and 17/24 (70.8%) grade IV gliomas. The overall 6-months PFS and one-year OS rates were 42% and 76%, respectively. The overall median PFS and OS time were 3 months (95% CI 2–15; 1–21) and 19 months (1–60), respectively.

### 3.2. Semiquantitative Analysis (n = 67)

**Figure 1:**
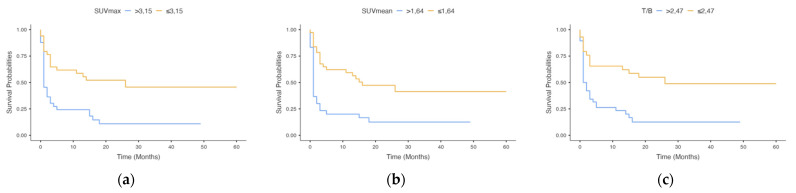
Kaplan–Meier estimates for progression-free survival by SUVmax (**a**), SUVmean (**b**) and T/B (**c**) (*p* < 0.001 in all parameters).

As reported in Table 2A, patients with recurrence within 6 months from MET-PET evaluation (*n* = 38) showed significantly higher SUVmax (*p* = 0.001), SUVmean (*p* = 0.002) and T/B values (*p* < 0.001) than patients without recurrence (*n* = 29). Table 3A reports the optimal threshold for SUVmax, SUVmean and T/B, along with the value of AUC, Youden’s index, sensitivity, specificity, negative and positive predictive values, and accuracy to predict the recurrence within 6 months from PET. Figure 1 reports the Kaplan–Meier progression-free survival curves for SUVmax, SUVmean and T/B ratio. Patients with SUVmax > 3.15, SUVmean > 1.64 and T/B > 2.47 had significantly worse PFS (*p* = 0.0019, *p* = 0.0001, *p* < 0.0011, respectively) than patients with lower levels of semiquantitative parameters.

**Figure 2:**
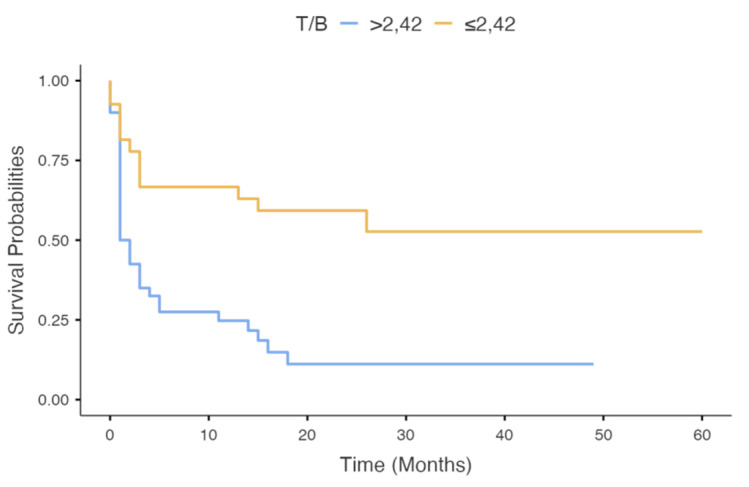
Kaplan–Meier estimates for overall survival by T/B (*p* = 0.031).

As reported in Table 2B, patients deceased after 12 months from PET evaluation (*n* = 16) showed significant higher SUVmax (*p* = 0.03), SUVmean (*p* = 0.03) and T/B values (*p* = 0.006) than alive patients (*n* = 51). Table 3B reports the optimal threshold for SUVmax, SUVmean and T/B, along with the value of AUC, Youden’s index, sensitivity, specificity, negative and positive predictive values, and accuracy to predict OS at 1 year from PET evaluation. Figure 2 reports the Kaplan–Meier overall survival curves for T/B ratio. Patients with T/B < 2.42 had better OS (*p* = 0.031) than patients with higher T/B value.

### 3.3. Kinetic Analyses (n = 46)

Table 4 reports clinical and metabolic parameters in patients (*n* = 46) who underwent dynamic MET-PET. At the time of last follow-up, 30/46 (65.2%) had recurrence (of whom 27 within 6 months and 3 after 6 months from MET-PET evaluation). Fourteen out of 46 (30.4%) patients died at last follow-up, all within 12 months from MET-PET.

**Figure 3:**
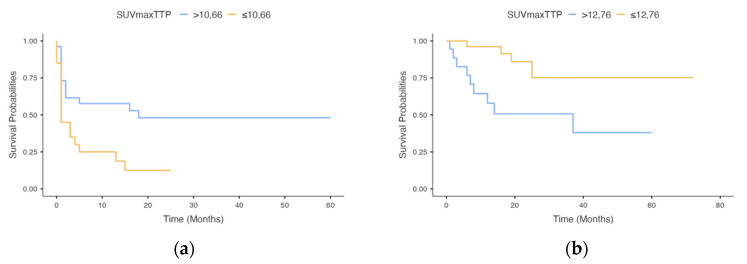
Kaplan–Meier estimates for progression-free survival (**a**) and overall survival (**b**) by SUVmax_TTP_ (*p* = 0.02 and *p* = 0.006, respectively).

Time–activity curve type A was observed in 16/27 (59.2%) and in 15/19 (78.9%) patients with and without recurrence within 6 months from PET evaluation, respectively (*p* = 0.1). As reported in Table 5A, patients with recurrence within 6 months from PET evaluation (*n* = 27) showed significantly higher SUVmax_TTP_ (*p* = 0.02) than patients who did not experience recurrence within 6 months (*n* = 19). No significant difference was found in the slope of the curve and TTP between recurrence and non-recurrence patients. Table 6A reports the optimal threshold for slope, TTP and SUVmax_TTP_, along with the value of AUC, Youden’s index, sensitivity, specificity, negative and positive predictive values, and accuracy to predict the recurrence within 6 months from PET. Figure 3a reports the Kaplan–Meier progression-free survival curves for SUVmax_TTP_. Patients with SUVmax_TTP_ > 10.66 had significantly worse PFS than patients with lower level of kinetic parameter (*p* = 0.02).

Time–activity curve type A was observed in 10/14 (71.4%) patients deceased within 12 months from MET-PET evaluation and in 21/32 (65.6%) still-living patients (*p* = 0.6). As reported in Table 5B, patients deceased within 1 year after PET evaluation (*n* = 16) showed significantly higher SUVmax_TTP_ (*p* = 0.01) than alive patients (*n* = 51). No significant difference was found in the slope of the curve and TTP between deceased and alive patients. Table 6B reports the optimal threshold for slope, TTP and SUVmax_TTP_, along with the value of AUC, Youden’s index, sensitivity, specificity, negative and positive predictive values, and accuracy to predict the recurrence within 6 months from PET. Figure 3b reports the Kaplan–Meier overall free survival curves for SUVmax_TTP_. Patients with SUVmax_TTP_ > 12.76 had significantly worse OS than patients with lower level of kinetic parameter (*p* = 0.006).

### 3.4. Univariate and Multivariate Survival Analysis

At univariate analysis, SUVmax, SUVmean, T/B and SUVmax_TTP_ were significant predictive factors for PFS (*p* = 0.02, *p* < 0.001, *p* = 0.002, *p* = 0.032, respectively); WHO grade at primary tumour, WHO grade at recurrence and SUVmax_TTP_ were significant predictive factors for OS (*p* = 0.001, *p* = 0.003, *p* = 0.02, respectively), as reported in Table 7.

At multivariate analysis, SUVmax_TTP_ was a significant independent predictor for PFS (HR 0.30 [0.09–1.04], *p* = 0.038); SUVmax_TTP_ tended to reach significance for OS (*p* = 0.058).

### 3.5. Subgroup Analysis of Patients with HGG (n = 40)

In the subgroup of histologically proven HGG (*n* = 40), we found that SUVmax, SUVmean, T/B and SUVmax_TTP_ were significant predictors of PFS (*p* = 0.012, *p* = 0.002, *p* < 0.001, *p* = 0.001 respectively). SUVmax, T/B and SUVmax_TTP_ were significant predictors for OS (*p* = 0.04, *p* = 0.01, *p* = 0.009, respectively).

**Figure 4:**
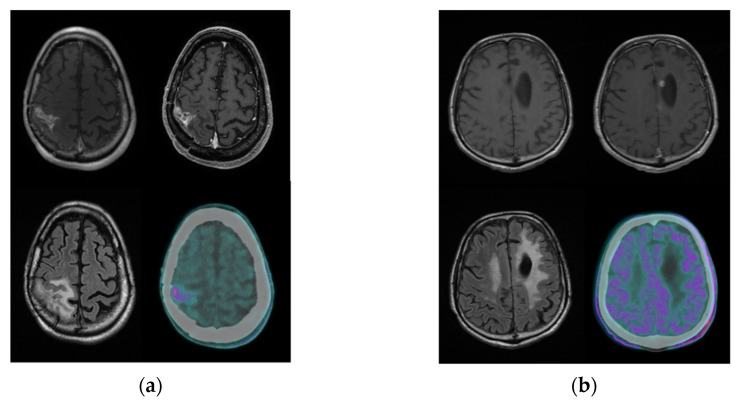
Representative examples of two patients with previous history of surgically treated grade IV HGG investigated with MET-PET scan for a suspicion of recurrence (T1-weighted, contrast-enhanced T1-weighted and FLAIR MR images and MET-PET images). In patient (**a**), MET-PET shows focal uptake higher than background in the right fronto-parietal region, corresponding to the site of abnormal signal intensity detected on MR and suspected for relapse; the T/B and SUVmax_TTP_ values were 3.94 and 15.58, respectively. Recurrence was confirmed by surgery 1 month after MET-PET evaluation, and the patient died 10 months later. In patient (**b**), the MET-PET scan did not show any area of focal uptake higher than background, especially in the area of abnormal signal intensity detected at MR on the left medial paratrigonal region and suspected for relapse; the T/B and SUVmax_TTP_ values were 1.45 and 8.64, respectively. The patient had no recurrence and was still alive at the time of the last follow-up.

Figure 4 reports MET-PET images of two representative glioma patients with suspected recurrence at MR.

## 4. Discussion

To the best of our knowledge, this is the first study that evaluated the potential role of the kinetic parameters extracted from ^11^C-methionine PET-CT dynamic acquisition, along with static parameters, in the prediction of clinical outcomes in patients with pre-treated low-grade and high-grade glioma, with MR suspected for recurrence during follow-up. The main finding of this study is that one parameter extracted from the time–activity curve (SUVmax associated with time-to-peak) of ^11^C-MET PET was a predictor of progression-free survival and overall survival in patients with suspected recurrence of glioma. Moreover, the static parameters evaluated, i.e., SUVmax, SUVmean and tumour-to-background ratio, may also identify those patients with a more favourable clinical course.

### 4.1. Detection of Glioma Recurrence and the Role of MET-PET

Aside from pilocytic astrocytoma, recurrence is awaited in any other astrocytoma and oligodendroglioma, with a tendency towards anaplastic transformation of LGGs. In fully treated glioblastoma, recurrence usually occurs between 7 and 9 months from surgery [37].

MR is the main technique in the evaluation of patients with glioma in each phase of the disease, including recurrence. However, scar tissue and post-radiation therapy changes (pseudo-progression or radiation necrosis) could affect its accuracy in detecting true recurrence, with consequent longer observation time and difficulty in therapeutic decision-making [38]. Moreover, no definitive MR-related prognostic factors are still used in clinical practice. Indeed, the only prognostic factors available for gliomas (including age, performance status, extent of primary resection and biomolecular markers: IDH status, 1p/19q co-deletion and the methylation of O6-methylguanine-DNA methyltransferase promoter metilation status) are obtained by the surgical sampling of primary glioma, and, since their stability over time is unclear, they are not capable of guiding therapeutic management during the course of the disease.

In cases of uncertain/suspected glioma recurrence at MR, early re-scan at 4–6 weeks is generally preferred over biopsy in clinical practice, while, when feasible, open surgery is generally preferred to simple biopsy in cases of gross recurrence. Clearly, glioma recurrence should be documented with a high level of accuracy by non-invasive techniques before performing a second surgery.

In this context, functional PET imaging with amino-acid radiotracers, including ^11^C-MET, proved to be a useful technique in the detection of glioma recurrence and in stratifying a subgroup of patients with glioma recurrence according to their prognosis [17,39,40,41,42]. In line with previous studies, our findings reinforce the role of MET-PET in the prognostication of glioma recurrence patients, proving for the first time a role of kinetic parameters in the prediction of prognosis of these difficult-to-manage patients. In this scenario, the availability of good imaging-based prognostic indices, such as static and dynamic PET parameters, could be very important in the actual clinical management of the disease, allowing the tailoring of the therapeutic strategy and, ultimately, the improvement of the prognosis of these patients.

### 4.2. Static MET-PET Parameters

From our data, all static ^11^C-MET parameters extracted from the ROI of the suspected lesion, including punctual numerical data (SUVmax and SUVmean) and fractal data (T/B ratio), were able to predict PFS, whereas only the T/B ratio was able to predict OS. Some papers have investigated the role of static MET-PET in the evaluation of prognosis in patients with suspected recurrence, with controversial results. Van Laereet al. [22], investigated the prognostic value of MET-PET and ^18^F-FDG in 30 patients with suspected recurrence of already-treated LGG and HGG; their data, in line with ours, showed that T/B with a cut-off of 2.2 was the best prognostic predictor of overall survival. Also de Witte et al. [19] studied the predictive value of static MET-PET in 85 patients with glioma before and after treatment: a significantly worse outcome was demonstrated when T/B was higher than 2.2 for a LGG and 2.8 for HGG. In our study, high T/B ratio was associated with poor prognosis in the overall population, as well as in the subgroup of HGGs. Contrarily, another study failed to prove a significant association between T/B and clinical outcome in patients with suspected glioma recurrence [43]. These conflicting results might be due to the inhomogeneity among studied characteristics, including glioma primary tumour and received treatment.

### 4.3. Kinetic MET-PET Parameters

Molecular imaging with dynamic PET allows the evaluation of the tracers’ kinetics in tissue and the quantification of the metabolic pathway of the radiotracer. Unlike static acquisition, dynamic acquisition allows the evaluation of the entire course of radioactivity from the vascular bed to the tissue molecular target as a function of time, building time–activity curves. The extrapolated kinetic parameters, allowing a better characterisation of the behaviour of tumour tissue over time, may be useful at different time-points of glioma history, including diagnosis/grading and recurrence detection, and for prognosis. Moreover, the dynamic acquisition of ^11^C-MET is not so demanding for patients, nor as time-consuming, lasting about 20 min (vs. 60 min for ^18^F-FET). Unlike fluorinated amino-acid radiotracers, kinetic ^11^C-MET parameters in glioma grading/diagnosis have been evaluated by few authors, finding controversial results [30,32,33]. As already pointed out, no data are available on the prognostic role of dynamic ^11^C-MET PET parameters in glioma recurrent patients.

Analysing the ^11^C-MET time–activity curves of our glioma recurrence patients, we found that neither the shape of the curve nor its slope were able to predict the prognosis either in the overall population or in the subgroup of HGGs. The prediction of prognosis using kinetic parameters in patients with suspected glioma recurrence has been recently investigated by Zaragori et al. [29] using ^18^F-DOPA. The authors failed to demonstrate a definitive role of kinetic parameters in the prediction of glioma recurrence/progression and survival, compare to static parameters. Indeed, the prognostic information was mostly achieved with conventional static parameters, with limited additional information provided by dynamic ones. Conversely, several studies have proven the usefulness of time–activity curves extracted from dynamic ^18^F-FET acquisition in different clinical contexts, including recurrence. Indeed, the curve shape/slope of ^18^F-FET has been associated with OS and PFS in patients with primary HGG [25] and LGG treatment-naïve patients [44], in patients with HGG recurrence before re-irradiation [27] and as diagnostic parameters in patients with primary or suspected recurrence of glioma [45,46]. A possible explanation regarding these different results in the same radiotracers’ category (amino-acids) in the same clinical contest (glioma recurrence) could be explained by the differences mechanism of uptake of these radiotracers.

Along with the shape and the slope of time–activity curves, we extrapolated two additional kinetic parameters from the time–activity curve, i.e., time-to-peak (TTP) and its associated SUVmax value (SUVmax_TTP_) [33]. From our data, SUVmax_TTP_ was the only kinetic parameter able to predict PFS and OS in both overall populations and in the HGGs subgroup. Moreover, this is the only PET parameter that turned out to be a prognostic factor for progression-free survival at multivariate analysis. Furthermore, this parameter almost reached a significant result at multivariate for OS (*p* = 0.058). It is important to underline that we analysed the data of the entire time–activity curve, including the first 5 min of acquisition, which is generally excluded from analysis. The early dynamic phase mainly reflects amino acid transport activity [47,48] and plays a crucial role in the characterisation of glioma. In fact, large amino acid transporter system is the principal mechanism for 11C-methionine uptake [49,50], and this uptake does not directly reflect the protein synthesis of the tissue, but it is considered to represent cell avidity for amino acid, which may be secondary to both cell proliferation and angiogenesis [51,52,53,54,55]. Interestingly, for the majority of our patients (40/46), TTP fell in the first 5 min of TAC. Our results allow us to speculate that the early phase could potentially identify a subpopulation at high risk of recurrence with higher accuracy than those parameters indicating only the tumour’s protein synthesis. We could hypothesise that an early dynamic acquisition lasting only 5 min could be enough to obtain essential prognostic information, avoiding a longer acquisition time.

### 4.4. Future Perspectives

According to our results of the kinetic analyses, future prospective studies investigating the relationship between kinetic parameters extrapolated from amino-acid PET-CT and perfusion imaging techniques, such as ^12^O-H_2_O PET and perfusion MR technics, are encouraged. Regarding perfusion MR imaging, a correlation between blood flows on arterial spin labelling MR and T/B on static MET-PET images has already been proven in recurrent glioblastoma [56]. In the last decade, the application of artificial intelligence and radiomics has gained increasing attention in medical imaging, including functional dynamic PET-CT. Indeed, the ability of principal component analysis to extract meaningful parametric maps from dynamic ^11^C-methionine PET-CT images has been recently investigated in glioma, highlighting the added value of dynamic over static PET acquisition in oncology [57].

Finally, it would be interesting to evaluate the real quantification of methionine metabolism into the tumour lesion. Indeed, the absolute quantification of methionine metabolism with a three-compartment kinetic model, calculating the full kinetic parameters (i.e., influx indices, the accumulation rate and the partition coefficient of methionine) [58,59] should allow the quantification of tumour protein synthesis and establish the actual consumption of ^11^C-methionine into the tumour. New diagnostic and prognostic kinetic parameters could be identified. To this aim, additional work is in progress.

### 4.5. Limitations

This study has several limitations, firstly including the retrospective nature and the related intrinsic patient selection. Indeed, patients included in this study have a long median interval (5 years) between primary diagnosis of the glioma to first MET-PET (i.e., time of suspected progressive disease), possibly reflecting the selection of patients with a better prognosis. Second, the relatively young mean age of the patients in our cohort, the heterogeneity of the tumours and treatments, at both diagnosis and recurrence, influences both PFS and OS. These aspects could also affect the tumour tissue behaviour at kinetic analyses, limiting the generalisation of our results. Nevertheless, the subgroup analysis performed on histologically proven HGG (at initial diagnosis and/or at second surgery) allows us to reduce the possible bias of tumour heterogeneity. Nonetheless, also the subgroup of adult GBM patients here considered had a rather long survival for the pathology (mean OS from surgery 36 months) and a rather young age (mean age 60). This may reflect a less aggressive or more chemo-/radio-sensitive pathology in these patients. Indeed, this series refers to tumours that had their histological diagnosis before publication of the new WHO 2016 and 2021 CNS tumour classifications [2,60], preventing us from analysing possible associations between MET-PET data and some of the currently known biomolecular prognostic factors, as IDH status, 1p/19q co-deletion and MGMT promoter metilation, ATRX mutation.

## 5. Conclusions

In patients with suspected glioma recurrence at MR, dynamic PET-CT with ^11^C-methionine was a useful diagnostic tool able to produce significant prognostic indices. In particular, both static and kinetic analyses produced a parameter (i.e., T/B and SUVmax_TTP_, respectively) able to predict both progression-free and overall survival in the whole population and in the high-grade glioma subgroup of patients. However, only SUVmax_TTP_, derived from dynamic acquisition and its relative kinetic analyses, resulted as a significant prognostic factor for progression free-survival at multivariate analyses. Future prospective PET-CT studies with more sophisticated analysis, including fully dynamic quantitative parameters or machine learning–driven approaches, are encouraged in order to confirm our preliminary data. Furthermore, new prognostic factors could be identified, able to give to clinicians pragmatic information in the management of these difficult-to-treat patients, allowing them to tailor the therapeutic strategy and, ultimately, improve the prognosis of these patients.

## Figures and Tables

**Table 1:** Patient clinical characteristics in the overall population (*n* = 67).

Characteristics	Number
patients	67
gender (M/F)	40/27
mean age ± SD (yrs)	48.4 ± 15.3
histopathology of primary tumour (*n*(%))	
pilocytic astrocitoma	1 (1.5%)
diffuse astrocitoma	12 (18%)
anaplastic astrocitoma	2 (3%)
ependimoma	1 (1.5%)
anaplastic ependimoma	2 (3%)
oligoastrocitoma	1 (1.5%)
anaplastic oligoastrocitoma	1 (1.5%)
oligodendroglioma	15 (22.3%)
anaplastic oligodendroglioma	7 (10.4%)
gliomatosi cerebri	1 (1.5%)
diffuse midline glioma	1 (1.5%)
glioblastoma	23 (34.3%)
WHO grade of primary tumour (*n*(%))	
I	1 (1.5%)
II	29 (43.3%)
III	13 (19.4%)
IV	24 (35.8%)
surgical approach of primary tumour (*n*(%))	
biopsy	8 (11.9%)
surgical resection	59 (88.1%)
first-line therapy following resection/biopsy (*n*(%))	
no therapy	12 (18%)
TMZ alone	8 (11.9%)
radiotherapy alone	7 (10.4%)
radiotherapy + TMZ	39 (58.2%)
n.a.	1 (1.5%)
recurrence (*n*(%))	46 (68.7%)
second therapy (after recurrence) (*n*(%))	
surgery	5 (11%)
surgery + radiotherapy + TMZ	5 (11%)
surgery + TMZ	3 (6.5%)
TMZ alone	13 (28.2%)
radiotherapy alone	6 (13%)
radiotherapy + TMZ	2 (4.3%)
no therapy	4 (8.7%)
n.a.	8 (17.4%)
death (*n*(%))	23 (34.3%)

SD: standard deviation; WHO: World Health Organisation; TMZ: temozolamide; n.a.: not applicable.

**Table 2:** Comparison of static MET-PET parameters (A) between patients with and without recurrence within 6 months from MET-PET; (B) between patients deceased and alive within 1 year from MET-PET scan.

A				B		
Static MET-PET Parameters	Recurrence within 6 Months (*n* = 38)	Nonrecurrence within 6 Months (*n* = 29)	*p*-Value	Deceased within 12 Months (*n* = 16)	Alive after 12 Months (*n* = 51)	*p*-Value
SUVmax	3.66 (2.76–5.10)	2.50 (2.09–3.23)	0.001	3.73 (3.05–5.25)	2.85 (2.13–3.69)	**0.032**
SUVmean	1.84 (1.36–2.29)	1.25 (1.02–1.61)	0.002	1.88 (1.60–2.28)	1.45 (1.04–1.97)	**0.036**
T/B	3.46 (2.51–4.59)	2.30 (1.84–2.86)	<0.001	3.95 (2.89–5.08)	2.47 (2.02–3.40)	**0.006**

Data are median (interquartile range). SUV: standardised uptake value; T/B: the ratio of SUVmax of tumour to SUVmean of background.

**Table 3:** Diagnostic performance of static MET-PET parameters assessed by additional receiver operating characteristic curve analysis using (A) progression-free survival at 6 months and (B) OS at 1 year as reference.

Static MET-PET Parameters		ROC Cut-Off	AUC	Younden’s Index	Sensitivity (%)	Specificity (%)	PPV (%)	NPV (%)	Accuracy
**A**	**SUVmax**	3.15	0.727	0.407	69.23	71.43	77.14	62.50	0.701
**SUVmean**		1.64	0.722	0.417	66.67	75.00	78.79	61.76	0.701
**T/B**		2.47	0.787	0.473	79.49	67.86	77.50	70.37	0.746
**B**	**SUVmax**	2.52	0.604	2.41	86.36	37.78	40.43	85.00	0.687
**SUVmean**		1.21	0.611	2.19	86.36	35.56	39.58	84.21	0.687
**T/B**		2.42	0.664	3.53	86.36	48.89	45.24	88.00	0.701

ROC: receiver operating characteristic; AUC: area under the curve; SUV: standardised uptake value; T/B: the ratio of SUVmax of tumour to SUVmean of background; PPV: positive predictive value; NPV: negative predictive value.

**Table 4:** Functional MET-PET parameters and main clinical characteristics of patients with dynamic acquisition (*n* = 46).

Characteristics	Number
Patients	46
Gender (M/F)	29/17
Mean age ± SD (yrs)	48.7 ± 15.7
WHO grade of primary tumour (*n*(%))	
I	1 (2.2%)
II	18 (39.1%)
III	7 (15.2%)
IV	20 (43.5%)
MET-PET positive (*n*(%))	30 (65.2%)
MET-PET negative (*n*(%))	16 (34.8%)
SUVmax (median (IQR))	3.35 (2.32–4.58)
SUVmean (median (IQR))	1.67 (1.03–2.04)
T/B (median (IQR))	2.88 (2.30–3.91)
Time–activity curve type A (increasing) (*n*(%))	31 (67.4%)
Time–activity curve type B (decreasing) (*n*(%))	15 (32.6%)
Slope (median (IQR))	9.66 × 10^−5^ (−2.45 × 10^−4^–1.33 × 10^−4^)
TTP (median (IQR)) in seconds	30 (20–35)
SUVmax_TTP_ (median (IQR))	11.5 (8.59–14.3)

SD: standard deviation; WHO: World Health Organisation; SUV: standardised uptake value; T/B: the ratio of SUVmax of tumour to SUVmean of background; TTP: time-to-peak.

**Table 5:** Comparison of kinetic MET-PET parameters (A) between patients with and without recurrence within 6 months from MET-PET; (B) between patients deceased and alive within 1 year from MET-PET scan.

A				B		
Kinetic MET-PET Parameters	Recurrence within 6 Months (*n* = 27)	Nonrecurrence within 6 Months (*n* = 19)	*p*-Value	Deceased within 12 Months (*n* = 14)	Alive after 12 Months (*n* = 32)	*p*-Value
**Slope**	9.87 × 10^−5^ (−2.91 × 10^−5^–1.36 × 10^−4^)	9.59 × 10^−5^ (1.00 × 10^−6^–1.15 × 10^−4^)	n.s.	1.03 × 10^−4^ (−2.55 × 10^−4^–2.07 × 10^−4^)	9.45 × 10^−5^ (−2.6 × 10^−5^–1.21 × 10^−4^)	n.s.
**TTP**	30 (20–35)	30 (22.5–42.5)	n.s.	25 (20–30)	32.5 (25–45)	n.s.
**SUVmax_TTP_**	12.80 (9.52–15.60)	9.25 (7.12–12.70)	0.02	13.09 (10.20–17.40)	10.20 (7.99–13.00)	0.01

TTP: time-to-peak; SUV: standardised uptake value; n.s.: not significant.

**Table 6:** Diagnostic performance of kinetic MET-PET parameters assessed by additional receiver operating characteristic curve analysis using (A) progression-free survival at 6 months and (B) OS at 1 year as reference.

Kinetic MET-PET Parameters		ROC Cut-Off	AUC	Younden’s Index	Sensitivity (%)	Specificity (%)	PPV (%)	NPV (%)	Accuracy
**A**	**Slope**	−5.65 × 10^−5^	0.521	0.250	84.21	40.74	50.00	78.57	0.587
**TTP**		40	0.519	0.131	31.58	81.48	54.55	62.86	0.609
**SUVmaxTTP**		10.66	0.678	0.335	70.37	63.16	73.08	60.00	0.674
**B**	**Slope**	−6.64 × 10^−4^	0.424	0.071	100.00	7.14	71.11	100.00	0.717
**TTP**		30	0.660	0.299	65.62	64.29	80.77	45.00	0.696
**SUVmax_TTP_**		12.76	0.710	0.433	71.43	71.88	52.63	85.19	0.783

ROC: receiver operating characteristic; AUC: area under the curve; PPV: positive predictive value; NPV: negative predictive value; TTP: time-to-peak; SUV: standardised uptake value.

**Table 7:** Prognostic factors related to progression-free survival (A) and overall survival (B) univariate and multivariate analyses.

A						B					
		Univariate Analysis		Multivariate Analysis				Univariate Analysis		Multivariate Analysis	
Variables	Number of Patients	HR (95% CI)	*p*	HR (95% CI)	*p*	Variables	Number of Patients	HR (95% CI)	*p*	HR (95% CI)	*p*
Age						Age					
>70	4	0.74 (0.23–2.41)	0.61			>70	4	0.41 (0.12–1.39)	0.1		
≤70	63					≤70	63				
Gender						Gender					
F	27	0.84 (0.44–1.58)	0.58			F	27	0.79 (0.34–1.82)	0.5		
M	40					M	40				
Grade at diagnosis						Grade at diagnosis					
LGG	30	1.77 (0.92–3.41)	0.09			LGG	30	11.10 (2.59–47.57)	0.001	0.92 (0.10–8.16)	0.9
HGG	37					HGG	37				
Grade at recurrence						Grade at recurrence					
HGG	40	0.52 (0.26–1.03)	0.06			HGG	40	0.11 (0.03–0.48)	0.003	0.98 (0.10–8.17)	0.9
LGG	27					LGG	27				
SUVmax						SUVmax					
>3.15	33	0.36 (0.19–0.70)	0.002	0.35 (0.05–2.22)	0.264	>2.52	46	0.45 (0.15–1.34)	0.1		
≤3.15	34					≤2.52	21				
SUVmean						SUVmean					
>1.64	30	0.28 (0.15–0.55)	<0.001	0.76 (0.16–3.52)	0.726	>1.21	46	0.41 (0.14–1.22)	0.1		
1.64	37					≤1.21	21				
T/B						T/B					
>2.47	38	0.32 (0.15–0.66)	0.002	0.53 (0.18–1.54)	0.244	>2.42	40	0.38 (0.14–1.03)	0.06		
≤2.47	29					≤2.42	27				
Curve Type						Curve Type					
A	31	1.89 (0.88–4.09)	0.1			A	31	0.80 (0.25–2.57)	0.7		
B	15					B	15				
Slope						Slope					
≤−0.0000565	15	0.53 (0.25–1.14)	0.1			≤−0.000664	2	0.64 (0.08–4.90)	0.6		
>−0.0000565	31					>−0–000664	44				
TTP						TTP					
≤40	36	0.82 (0.31–2.17)	0.68			≤30	27	0.31 (0.09–1.12)	0.07		
>40	10					>30	19				
SUVmax_TTP_						SUVmax_TTP_					
>10.66	25	0.41 (0.18–0.93)	0.032	0.30 (0.09–1.04)	0.038	>12.76	18	0.28 (0.09–0.83)	0.022	0.30 (0.09–1.04)	0.058
≤10.66	21					≤12.76	28				

LGG: low-grade glioma; HGG: high-grade glioma; SUV: standardized uptake value; T/B: the ratio of SUVmax of tumour to SUVmean of background; TTP: time-to-peak.

## Data Availability

The data presented in this study are available on request from the corresponding author.
